# Supplementary material for: Modeling glioblastoma heterogeneity as a dynamic network of cell states
Source: Mol Syst Biol. 2021 Sep 16;17(9):e10105. doi: 10.15252/msb.202010105 (PMC8444284; doi:10.15252/msb.202010105)
Supplement: Supplementary file 6 — Source Data for Figure 5 [file MSB-17-e10105-s004.zip › Figure5A_sourcedata/GSEA_3071/nosigdb_stateIV.GseaPreranked.1622795164626/NB_KI_CLUSTER18.html]

Details for gene set NB\_KI\_CLUSTER18[GSEA]

|  || Dataset | state53071 |
| Phenotype | NoPhenotypeAvailable |
| Upregulated in class | na\_neg |
| GeneSet | NB\_KI\_CLUSTER18 |
| Enrichment Score (ES) | -0.652177 |
| Normalized Enrichment Score (NES) | -3.1487675 |
| Nominal p-value | 0.0 |
| FDR q-value | 0.0 |
| FWER p-Value | 0.0 |
Table: GSEA Results Summary

  

Fig 1: Enrichment plot: NB\_KI\_CLUSTER18      
 Profile of the Running ES Score & Positions of GeneSet Members on the Rank Ordered List

  

| PROBE | GENE SYMBOL | GENE\_TITLE | RANK IN GENE LIST | RANK METRIC SCORE | RUNNING ES | CORE ENRICHMENT || 1 | LHFPL6 |  |  | 52 | 1.815 | 0.0103 | No |
| 2 | LIMA1 |  |  | 58 | 1.805 | 0.0296 | No |
| 3 | COL1A2 |  |  | 137 | 1.425 | 0.0306 | No |
| 4 | A2M |  |  | 188 | 1.283 | 0.0353 | No |
| 5 | RGS16 |  |  | 237 | 1.178 | 0.0393 | No |
| 6 | FCGRT |  |  | 343 | 0.979 | 0.0302 | No |
| 7 | FOS |  |  | 530 | 0.786 | 0.0033 | No |
| 8 | ADAMTS1 |  |  | 875 | 0.570 | -0.0562 | No |
| 9 | SH3BGRL |  |  | 1001 | 0.522 | -0.0743 | No |
| 10 | C1R |  |  | 1072 | 0.502 | -0.0821 | No |
| 11 | MFGE8 |  |  | 1087 | 0.496 | -0.0792 | No |
| 12 | TNFRSF1A |  |  | 2234 | 0.264 | -0.2958 | No |
| 13 | MYLK |  |  | 2532 | -0.273 | -0.3496 | No |
| 14 | ITGB1 |  |  | 2817 | -0.308 | -0.4005 | No |
| 15 | SOCS3 |  |  | 3148 | -0.353 | -0.4598 | No |
| 16 | COL18A1 |  |  | 3266 | -0.370 | -0.4781 | No |
| 17 | STOM |  |  | 3448 | -0.400 | -0.5083 | No |
| 18 | LGALS1 |  |  | 3692 | -0.443 | -0.5499 | No |
| 19 | CD99 |  |  | 3752 | -0.456 | -0.5561 | No |
| 20 | TPM4 |  |  | 3761 | -0.458 | -0.5525 | No |
| 21 | CALD1 |  |  | 3763 | -0.458 | -0.5476 | No |
| 22 | MEF2C |  |  | 3894 | -0.485 | -0.5670 | No |
| 23 | NUPR1 |  |  | 4156 | -0.556 | -0.6108 | No |
| 24 | VIM |  |  | 4255 | -0.582 | -0.6231 | No |
| 25 | IGFBP5 |  |  | 4280 | -0.592 | -0.6210 | No |
| 26 | IGFBP7 |  |  | 4331 | -0.607 | -0.6238 | No |
| 27 | YBX3 |  |  | 4405 | -0.637 | -0.6307 | No |
| 28 | LGALS3 |  |  | 4418 | -0.641 | -0.6258 | No |
| 29 | CYR61 |  |  | 4498 | -0.678 | -0.6334 | No |
| 30 | CST3 |  |  | 4577 | -0.723 | -0.6402 | No |
| 31 | THY1 |  |  | 4587 | -0.729 | -0.6338 | No |
| 32 | FILIP1L |  |  | 4671 | -0.778 | -0.6410 | No |
| 33 | RRBP1 |  |  | 4672 | -0.779 | -0.6323 | No |
| 34 | ID3 |  |  | 4686 | -0.788 | -0.6259 | No |
| 35 | MGP |  |  | 4824 | -0.890 | -0.6422 | Yes |
| 36 | SERPINH1 |  |  | 4829 | -0.895 | -0.6330 | Yes |
| 37 | CEBPB |  |  | 4835 | -0.902 | -0.6238 | Yes |
| 38 | NR2F2 |  |  | 4870 | -0.937 | -0.6199 | Yes |
| 39 | ITGA1 |  |  | 4951 | -1.019 | -0.6238 | Yes |
| 40 | ZFP36L1 |  |  | 4969 | -1.040 | -0.6154 | Yes |
| 41 | NOTCH3 |  |  | 5003 | -1.098 | -0.6095 | Yes |
| 42 | UACA |  |  | 5013 | -1.113 | -0.5987 | Yes |
| 43 | CYTOR |  |  | 5037 | -1.167 | -0.5901 | Yes |
| 44 | CAVIN1 |  |  | 5050 | -1.185 | -0.5791 | Yes |
| 45 | MT2A |  |  | 5061 | -1.216 | -0.5675 | Yes |
| 46 | TIMP1 |  |  | 5086 | -1.257 | -0.5580 | Yes |
| 47 | GADD45B |  |  | 5099 | -1.292 | -0.5459 | Yes |
| 48 | TGFBI |  |  | 5104 | -1.305 | -0.5320 | Yes |
| 49 | LMNA |  |  | 5116 | -1.325 | -0.5193 | Yes |
| 50 | MYH9 |  |  | 5132 | -1.366 | -0.5069 | Yes |
| 51 | PDLIM1 |  |  | 5142 | -1.392 | -0.4931 | Yes |
| 52 | SERPINF1 |  |  | 5148 | -1.414 | -0.4782 | Yes |
| 53 | BGN |  |  | 5160 | -1.486 | -0.4637 | Yes |
| 54 | AXL |  |  | 5165 | -1.518 | -0.4475 | Yes |
| 55 | PRSS23 |  |  | 5179 | -1.562 | -0.4325 | Yes |
| 56 | IGFBP4 |  |  | 5211 | -1.701 | -0.4194 | Yes |
| 57 | TFPI |  |  | 5212 | -1.705 | -0.4003 | Yes |
| 58 | COL4A2 |  |  | 5235 | -1.917 | -0.3831 | Yes |
| 59 | TPM2 |  |  | 5242 | -1.971 | -0.3622 | Yes |
| 60 | PDGFRB |  |  | 5253 | -2.141 | -0.3402 | Yes |
| 61 | COL6A2 |  |  | 5258 | -2.256 | -0.3157 | Yes |
| 62 | COL4A1 |  |  | 5270 | -2.478 | -0.2901 | Yes |
| 63 | MYL9 |  |  | 5272 | -2.509 | -0.2622 | Yes |
| 64 | COL6A1 |  |  | 5278 | -2.689 | -0.2331 | Yes |
| 65 | CAV1 |  |  | 5281 | -2.850 | -0.2016 | Yes |
| 66 | FN1 |  |  | 5284 | -3.017 | -0.1683 | Yes |
| 67 | TAGLN |  |  | 5285 | -3.021 | -0.1345 | Yes |
| 68 | COL3A1 |  |  | 5287 | -3.508 | -0.0954 | Yes |
| 69 | COL6A3 |  |  | 5288 | -3.654 | -0.0546 | Yes |
| 70 | COL1A1 |  |  | 5290 | -4.897 | -0.0000 | Yes |
Table: GSEA details [plain text format]

  

Fig 2: NB\_KI\_CLUSTER18: Random ES distribution      
 Gene set null distribution of ES for **NB\_KI\_CLUSTER18**
